# Development and effects of a high-risk pregnancy emotive role-play program for nursing students: a quasi-experimental study

**DOI:** 10.4069/kjwhn.2022.12.06

**Published:** 2022-12-29

**Authors:** Bo Gyeong Lee, Sun-Hee Kim

**Affiliations:** College of Nursing, Research Institute of Nursing Science, Daegu Catholic University, Daegu, Korea

**Keywords:** Communication, Emotional intelligence, Nursing students, Role playing, Work performance

## Abstract

**Purpose:**

This study aimed to develop an emotive role-play program for nursing students focusing on high-risk pregnancy and analyze its effects on communication skills, clinical performance, and emotional intelligence.

**Methods:**

A quasi-experimental nonequivalent comparison group design was adopted with 83 nursing students (experimental group, 45; comparison group, 38) who participated voluntarily in an extracurricular program. The preliminary survey was conducted on November 3 and November 4, 2020, and the follow-up survey was conducted on November 12, 2020, for the comparison group and on November 27, 2020, for the experimental group. A program that included five role-play scenarios related to induced labor, preeclampsia, premature rupture of membranes, preterm labor, and infertility was developed by a group of experts and presented to the experimental group over 11 total hours across 3 days. Each student participated in a role-play scenario as a patient, family member, or nurse and observed three other scenarios. The comparison group received a workbook after the follow-up evaluation. The independent t-test was performed to analyze changes in communication skills, clinical performance, and emotional intelligence.

**Results:**

Communication skills (t=1.84, *p*=.035) and clinical performance (t=2.75, *p*=.004) significantly increased in the experimental group compared to the comparison group. A significant difference was not observed between the experimental and comparison groups for emotional intelligence (t=1.36, *p*=.088).

**Conclusion:**

The emotive role-play program concerning high-risk pregnancy was effective in improving nursing students’ communication skills and clinical performance and can be used in nursing education related to high-risk pregnancy and childbirth.

## Introduction

High-risk pregnancy refers to a physiological and psychological condition that increases the likelihood of endangering the health or life of the fetus and mother [1]. Approximately 15% of all pregnancies are high-risk, and high-risk pregnancies are expected to become more common due to late pregnancy and childbirth in Korea’s low birthrate era [2,3]. Therefore, there is an urgent need for healthcare professionals who can offer expert services that can improve the health outcomes of high-risk pregnant women.

High-risk pregnant women have been reported to experience high levels of anxiety [1,4,5], stress [1,5], and depression [1,4]. For example, in induced labor, healthcare providers often struggle to predict the progress of delivery when uterine contractions are artificially induced by uterine stimulants. Along with extreme pain, women in labor experience fear and anxiety about surgery and complications following failed induced labor [6,7]. In addition, women suffering from preterm labor, premature rupture of membranes, and preeclampsia feel psychological pressure to extend the pregnancy period and also experience high anxiety due to concerns about their own health, as well as fetal well-being [8-10]. Infertile women, another high-risk group, experience stress and depression due to the loss of opportunities for pregnancy, the risk of pregnancy failure, and prolonged social isolation [11]. As such, high-risk pregnancy causes stress, anxiety, helplessness, and uncertainty, which are related to obstetric complications, such as hypertension, preeclampsia, dystocia, and low birth weight, and can negatively impact the maternal-fetal relationship. Therefore, psychological interventions are crucial for high-risk pregnant women [5,12].

Emotional intelligence refers to the ability to recognize one’s own and others’ emotions, distinguish between physical and mental reactions, and use one’s judgment to interact with the thoughts and actions of others [13]. Emotional intelligence is an important competency for healthcare providers in treating and caring for high-risk pregnant women since it enables appropriate responses to others’ emotional needs and can enhance the physical and psychological well-being of patients [14]. Emotional intelligence can be improved through continuing training and education [15] and would benefit nursing students as they learn how to become nurses.

Furthermore, nursing students must have strong interpersonal skills to manage the needs of the patients they encounter during clinical practicum [16] and improving communication skills to form strong interpersonal relationships with patients is important [17]. Nevertheless, due to the lecture-oriented approach in theoretical education and observation-oriented approach in clinical practicum, nursing students often lack sufficient educational opportunities to acquire strong patient communication skills [18]. Thus, they may experience difficulties forming therapeutic relationships with patients as nurses [15]. Since well-grounded communication skills are essential for forming therapeutic trust between nurses and patients as well as patients’ families [18], this underscores the need for creative ways to strengthen nursing students’ communication competency.

Additionally, nursing students need continuous clinical performance training in their courses to improve their adaptability to actual nursing situations and professionalism [19]. Clinical performance refers to the ability to adopt appropriate nursing practices in clinical settings by applying one’s knowledge, skills, attitudes, and judgments [20]. Role-play is widely used in nursing education as a tool to improve clinical performance and communication skills.

Role-play is an experiential learning strategy in which learners actively participate in scenarios to understand the intended learning outcomes and receive feedback. Role-play has been recognized as a useful strategy for developing communication and clinical performance skills of health care providers [21,22]. In the field of women’s health, few studies have used role-play and analyzed its effects; however, many previous studies have evaluated the effects of role-play programs for patients with mental illness [23], terminal cancer [24], and arthritis [25], as well as emergency room patients [26]. In these studies, role-play improved nursing students’ nursing performance [26,27], communication skills [23-25,28], emotional intelligence [28], and knowledge and skills [24,26,29]. During role-play, participants can explore the clients’ emotional realm and experience others’ emotions in realistic scenarios [30,31]. In this sense, role-play based on emotional intelligence, which focuses on strengthening empathy for and communication with clients, can be useful in learning how to care for and communicate with high-risk pregnant women. Therefore, in this study, we used role-play as a method to improve the emotional intelligence of nursing students in caring for women with high-risk pregnancies.

Our review of previous studies in South Korea (hereafter, Korea) and overseas on the effects of role-play programs for nursing students showed only a few that focused on the relationships between nurses and patients in the field of women’s health [32,33]. In particular, few studies have examined the effects of role-play programs related to nurses’ relationships with high-risk pregnant women. Therefore, this study aimed to develop an emotive role-play program focused on high-risk pregnant women to improve nursing students’ communication skills, clinical performance, and emotional intelligence; also, to evaluate the program’s effectiveness. Therefore, the hypotheses of this study were as follows. First, we hypothesized that the change in communication skills after the application of the program would be greater in the experimental group than in the comparison group. Second, the change in the clinical performance of caring for high-risk pregnant women (hereafter, clinical performance) would be greater in the experimental group than in the comparison group. Third, compared to the comparison group, the experimental group would show a greater change in emotional intelligence after the program.

## Methods

**Ethics statement:** This study was approved by the Institutional Review Board of Daegu Catholic University (CUIRB-2019-0077). Informed consent was obtained from the participants by an independent research assistant, and all students were assured that their participation was strictly voluntary.

### Study design

This was a quasi-experimental nonequivalent comparison group pretest-posttest study to evaluate the effects of an emotive role-play program for nursing students focused on caring for high-risk pregnant women. The study was described according to the TREND (Transparent Reporting of Evaluations with Nonrandomized Designs) reporting guidelines [34].

### Development of a high-risk pregnancy emotive role-play program

As the first step of developing a high-risk pregnancy emotive role-play program for nursing students, expert consultation was sought from three professors in women’s health in nursing and three delivery room nurses. They were asked to select four high-risk pregnancy and childbirth-related health problems for which they felt the strongest need and greatest importance in clinical practice. As a result, five areas were identified: induced labor, preeclampsia, preterm premature rupture of membranes, preterm labor, and infertility. The experts consulted were on average 40.8 years of age, had nursing education experience of 4.5 years, and had clinical experience in pregnancy and childbirth for 10.3 years. Among them, two delivery room nurses developed a role-play scenario for nursing students based on real clinical cases. The authors then modified the scenario and finalized it after reviewing and discussing it with the two nurses who had originally developed it.

Based on the nine-session role-play model of teaching [35], the researchers designed the program, modified as follows: session 1 for introduction, sessions 2 and 3 for preparation, session 4 for role-play training, session 5 for performing and watching role-play scenarios, and session 6 for evaluation and feedback. Among the nine sessions of the role-play class model [32], “focusing the attention of the group” was incorporated into the introduction session; “selection of participants to select actors,” “preparation in advance,” and “preparation of observers” were incorporated into the preparation session; “performance” and “discussion and evaluation of important points” were incorporated into the role-play training session; “reacting” was incorporated into performing and watching role-play scenarios; and “discussion and evaluation” and “exchange of experiences” were incorporated into evaluation and feedback.

In the final program, the introduction session (session 1, 30 minutes) consisted of welcoming the participants, providing an introduction to the program, and drafting participants’ pledge of sincere participation, respect for humanity, and confidentiality. The preparation sessions (sessions 2 and 3) included a workshop (session 2, 2.5 hours) on communication skills for nurses when providing emotional nursing care for pregnancy and delivery; and a session (session 3, 2 hours) to hear stories from women who experienced induced labor, preeclampsia, preterm premature rupture of membranes and preterm labor, and infertility. Role-play training was done in session 4 (2 hours) and included group role-plays script writing and practice. Session 5 (3 hours) watching role-plays and session 6 (1 hour) was evaluation and feedback, which included sharing experiences, evaluation, and wrap-up (Table 1).

### Testing the high-risk pregnancy emotive role-play program

#### Participants

**Figure 1.**
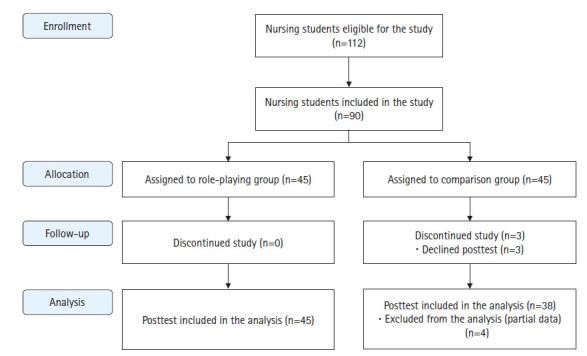
Flow diagram of the study design.

The participants in this study were third-year nursing students enrolled at the researchers’ institution who provided written informed consent to participate in the study. The number of participants required for this study was calculated using G*Power 3.1.2. Since the main effect variable was communication skills, considering the effect sizes of previous studies on communication skills [15,23,36], the minimum number of samples required per group was 29 when calculated using an effect size of 0.67, a significance level of .05, and a power of .80 for the independent t-test. Considering a possible dropout rate of 35%, a total of 90 participants (45 per group) was set as the target number of study participants. After posting a recruitment announcement on the campus, an assistant researcher explained the purpose and methods of the study to the first 90 students who expressed interest in participating. Among the first 90 applicants, those who were able to follow the program schedule were assigned to the experimental group, while those who were unable to accommodate the schedule were assigned to the comparison group. After excluding the data from seven dropouts in the comparison group, data from 45 participants in the experimental group and 38 participants in the comparison group were used for the final analysis (Figure 1).

#### Study procedures

A preliminary survey to evaluate the effects of the high-risk pregnancy emotive role-play program was conducted on November 3 and November 4, 2020, for both the experimental and comparison groups. Research assistants independent of the intervention program distributed questionnaires to all students who chose to voluntarily participate in an empty classroom 1 week before the program. The students took approximately 10 to 15 minutes to complete the preliminary survey questionnaire. To avoid the spread of information, the follow-up survey of the comparison group was completed on November 12, 2020, before the intervention program for the experimental group had begun. For the experimental group, the follow-up survey was completed on November 27, 2020, immediately after the end of the program. Each participant in both groups was provided with a small gift (worth approximately 4 US dollars) upon completing and submitting the questionnaire.

#### Experimental treatment

The experimental group participated in the program, which was conducted for a total of 11 hours across 3 days in a room modeled as a clinical setting and a lecture room. The participants were divided into eight groups of five or six students. On the first day, in session 1, an introduction to the program and participants was provided; and in session 2 (the first part of the preparation stage), the director of the communication center delivered a lecture on the importance of communication and communication methods using examples from pregnancy- and childbirth-related scenarios. This was followed by group activities and student presentations about communication-related topics. On the second day, in session 3 (the second part of the preparation stage), four women shared their high-risk pregnancy experiences, and in session 4, each group randomly reviewed two scenarios from one of the following nine scenarios of five themes: induced labor (3 scenarios), preeclampsia (3 scenarios), preterm premature rupture of membranes and premature labor (2 scenarios), and infertility (1 scenario). Each group then selected one case for script modification and role assignment. Ultimately the nursing scenarios were for women with induced labor (two groups), preeclampsia (three groups), preterm premature rupture of membranes and preterm labor (two groups), and infertility (one group). Each group received directing and acting lessons from a theater actor. After giving the groups one week to practice the role-play scenario, sessions 5 and 6 were conducted on the third day of the program to perform and observe the role-play scenarios and provide evaluation and feedback. After a rehearsal, role-play scenarios with the same theme (e.g., preeclampsia scenario in three groups) were performed simultaneously during session 5, and those who were not performing at the time were asked to observe the other groups’ performances; thus, each student participated in one role-play scenario and observed three other role-play scenarios. Each role-play scenario lasted approximately 30 minutes. Students in the experimental group recorded a reflection diary for each activity across the 3 days of participating in the program. After performing the role-play scenarios in session 6, students gathered in a classroom to share their experiences and feelings and evaluate the role-play performances (Table 1). The comparison group did not attend any intervention and received the same workbook as the experimental group after the follow-up survey.

### Measurements

In the preliminary and follow-up surveys, communication skills, clinical performance, and emotional intelligence were assessed.

#### Communication skills

Communication skills were assessed using a tool modified and supplemented by Yoon [37] based on the items used in Kim’s study (unpublished data). The tool contains 10 subdomains related to communication skills, including noticing, participating, sharing, active listening, accompanying, complimenting, comforting, hoping, forgiving, and accepting. The 50-item tool contains five questions on each subdomain, with each rated on a 5-point Likert scale (from “very little,” 1 to “very much,” 5). In the study by Yoon [37], the point-average scores for each of the 10 subdomains were used; however, in the current study, the item mean scores were used with higher scores indicating stronger communication skills. At the time of the development of the tool, Cronbach’s α was .95 [37]; in our study, it was .97.

#### Clinical performance of nursing care for high-risk pregnant women

The 19-item tool developed by Yang and Park [38] based on the clinical performance tool designed by Lee et al. [39] was used after obtaining approval for its use and modification. Some items were modified to better cover the topic of nursing care for high-risk pregnant women, e.g., “I can perform nursing process to solve nursing problems for high-risk pregnant women,” “I can conduct effective education suitable for high-risk pregnant women.” The tool has six subdomains: nursing process (four items), nursing intervention (four items), psychosocial nursing (three items), education (three items), physical assessment and patient monitoring (two items), and basic nursing (three items). Each item is answered on a 5-point Likert scale (from “not at all,” 1 to “very much so,” 5). The point-average score was calculated in this study, the item mean scores were used with higher scores indicating better clinical performance. Cronbach’s α was .96 at the time of development by Lee et al. [39], .86 in Yang and Park’s study [38], and .95 in the current study.

#### Emotional intelligence

Emotional intelligence was assessed using the Emotional Intelligence Scale developed by Wong and Law [40] that was adopted by Jung [41] after securing approval from the developers for its use. The tool consists of a total of 16 items across four subdomains: understanding one’s own emotions (four questions), understanding others’ emotions (four questions), comparisonling emotions (four questions), and use of emotions (four questions). Each item is rated on a 7-point Likert scale (from “not at all,” 1 to “very much so,” 7). The point-average score was calculated in this study, the item mean scores were used with higher scores indicating greater emotional intelligence. Cronbach’s α was .87 at the time of development [40] and was .93 in the current study.

#### General characteristics

For general characteristics, students’ age, sex, and religion were asked in the preliminary survey.

### Data analysis

All data in this study were analyzed using IBM SPSS for Windows ver. 25.0 (IBM Corp., Armonk, NY, USA). Frequencies and percentages were used for the general characteristics of the participants. The chi-square test, Fisher exact test, and t-test were used to test the homogeneity of the two groups’ communication skills, clinical performance, and emotional intelligence. The independent t-test was used to compare differences in the changes in communication skills, clinical performance, and emotional intelligence between the experimental and comparison groups. The normality of the measured variables was confirmed using the Shapiro-Wilk test (pretest and posttest). There were no missing data for the main variables and general characteristic variables, and the statistical significance level of the analysis data was .05.

## Results

### Homogeneity testing for general characteristics, communication skills, clinical performance, and emotional intelligence

The average age of the experimental group was 21.24±1.05 years, and 21.55±1.18 years for the comparison group. Most participants were women (44 in the experimental group [97.8%] and 34 in the comparison [89.5%]) and many had no religion (27 in the experimental group [60.0%] and 29 in the comparison [76.3%]) (Table 2). Age (*p*=.211), sex (*p*=.174), and religion (*p*=.114) were not significantly different between the groups, indicating homogeneity.

In the preliminary survey, for the experimental group the average communication skills score was 4.01±0.41 points, while the average clinical performance score was 3.70±0.48 points and the average emotional intelligence score was 5.32±0.74 points. The comparison group had average scores of 4.08±0.49 points for communication skills, 3.80±0.51 points for clinical performance, and 5.24±0.83 points for emotional intelligence. Communication skills (*p*=.499), clinical performance (*p*=.396), and emotional intelligence (*p*=.608) were not significantly different between the groups, indicating homogeneity (Table 2).

### Testing the effects of the high-risk pregnancy emotive role-play program

The average change in communication skills in the experimental group after the program was 0.20±0.40 points, which was statistically significantly greater than the average change of 0.04±0.37 points in the comparison group (t=1.84, *p*=.035). Therefore, the first hypothesis was supported (Table 3).

The average change in the clinical performance in the experimental group after the program was 0.52±0.50 points, which was statistically significantly greater than the average change of 0.21±0.51 points in the comparison group (t=2.75, *p*=.004). Therefore, the second hypothesis was also supported (Table 3).

The average change in emotional intelligence in the experimental group after the program was 0.47±0.77 points, while it was 0.25±0.69 points in the comparison group. Although communication skills increased more in the experimental group, no statistically significant difference was observed (t=1.36, *p*=.088). Therefore, the third hypothesis was not supported (Table 3).

## Discussion

In this study, the communication skills of the experimental group showed a significant increase compared to the comparison group after the implementation of the emotive role-play program. A direct comparison is difficult due to the lack of studies examining the effect of role-playing on the relationships between nursing students and patients in the field of women’s health nursing. However, a study by Seo and Jeong [23] showed that a role-play program for nursing students on psychological nursing care resulted in significant improvement in communication skills. Bosse et al. [42] also examined the effect of a role-play program for medical students and reported that compared to a comparison group who received the existing curriculum, the experimental group that participated in the role-playing program had higher self-efficacy in terms of their communication skills with pediatric patients and their parents, showing similar results to those of our study. Judging from these results, role-play programs can be regarded as a useful educational method to improve nursing students’ communication skills. In recent years, simulation-based education programs using high-fidelity simulators have been actively used in nursing education; however, these programs have limitations such as high initial costs, difficulties in operating equipment, and providing training for instructors [43,44]. In addition, while many studies have consistently reported improved nursing knowledge, critical thinking, and clinical performance through high-fidelity simulation-based education programs [44-46], their effects on communication skills have not been extensively investigated, and the few results in this area have been inconsistent. For example, no significant difference in communication skills was observed between an experimental group and a comparison group that participated in a simulation-based program [46]. As such, role-play programs may be a more feasible and effective alternative for strengthening nursing students’ communication competency, especially in terms of their relationships with patients who require psychological support and intervention. Therefore, role-play programs can be actively used as a teaching method for strengthening the communication competency of nursing students in caring for high-risk pregnant women.

The finding that clinical performance in the experimental group significantly improved is also similar to prior studies. In a study by Bosse et al. [42], in addition to communication skills, the objective structured clinical examination score of the experimental group that participated in role-play scenarios was significantly improved compared to that of the comparison group. In addition, according to a study by Heidarzadeh et al. [26], education on an emergency patient classification system conducted with nursing students using role-play scenarios resulted in significant improvement in applying the classification system, compared to the group who followed the traditional curriculum. Based on these results, role-play can likely be widely adopted not only to strengthen students’ communication competency but also to improve various clinical performance skills. To overcome some of the limitations of simulators, such as difficulties with communication between nursing students and patients/families, a hybrid program that combines simulation-based training and standardized patients has been proposed as an alternative [44,47]. According to previous studies, these measures have a positive effect on nursing students’ communication skills and the formation of therapeutic relationships with patients and family members [44,47]. Future studies that develop and analyze the effectiveness of a program that combines simulators and role-play scenarios are needed to improve our understanding.

In this study, emotional intelligence did not improve significantly compared to the comparison group after implementing the program, which contrasts with the literature. In a previous study by Oh and Kim [48], an emotive intelligence-based interpersonal relationship training program was conducted with freshmen at a nursing school, and the average emotional intelligence score was found significantly improve among those in the experimental group compared to the comparison group. In addition, a study of an emotional intelligence improvement program for nursing students found that the average emotional intelligence score of the experimental group significantly improved compared to the comparison group [15], which contrasts with our study findings. This difference may be due to diffusion of the experimental effect; since this study was conducted during the semester at a single university and with students in the same grade, despite our efforts to comparison for diffusion between the groups, it may have been difficult to completely exclude its effects. Furthermore, as the emotional intelligence scores at baseline were relatively high, a ceiling effect may be considered. In Lee and Gu’s study [15], the same measurement tool was used as our study to assess emotional intelligence and the baseline average scores (3.40±0.41 for the experimental group, 3.25±0.44 points for the comparison group) were lower than our study. The emotional intelligence scores of the experimental group, which were measured twice after the program (3.95±0.41 and 3.80±0.40 points) were also both lower than our students’ average score. Similar results were also found in other studies that measured nursing students’ emotional intelligence using the same tool. Kim [49] analyzed the effects of nursing students’ metacognition and emotional intelligence on self-leadership and reported an average emotional intelligence score of 3.63±0.48 points and Shin and Lee’s [50] study on emotional intelligence according to enneagram personality type reported an average of 4.70±0.75 points, which are both slightly lower than that of the participants in our study. This is likely due to differences in the nursing students’ experience levels as most of the research participants in prior studies were freshmen or students in earlier stages of their nursing program who had not yet developed clinical experience, whereas the participants in our study were third-year students with direct or indirect exposure to caring for high-risk pregnant women from the previous semester through a clinical practicum. Therefore, the participants in our study were likely better able to understand and sympathize with the physical and psychological pain of high-risk pregnant women. Therefore, emotive role-play programs for nursing care of high-risk pregnant women may be more effective when provided for nursing students in earlier curricular stages.

The duration and intensity of the intervention may have also played a role in outcome differences. While our program was conducted on an intensive timeline over a short period (a total of 11 hours across 3 days), in Oh and Kim’s study [48], which observed significant improvement in emotional intelligence, the program was conducted in weekly 100-minute sessions over a total of 8 weeks. In Lee and Gu’s study [15], the program was conducted in eight sessions for a total of 20 hours across 4 weeks. In other words, our study examined the possibility of using a short-term intensive role-play program as a way to improve communication skills and clinical performance. However, given that the short period may have been limited for improving emotional intelligence, such programs should be supplemented with routine, long-term training and further tested in the future. In addition, no unintended effects of the intervention program were observed in this study.

Since this study was conducted with nursing students from a single university, there are limitations in applying study findings to all nursing students. In addition, since the study was conducted during the semester with third-year students at the same university, the effect of diffusion cannot be completely ruled out. Nevertheless, this study is significant as the first to our knowledge, to develop a role-play program focused on high-risk pregnancy and to test its effectiveness in improving nursing students’ emotional intelligence and communication competency. In addition, our study confirmed the effectiveness of role-play programs as a feasible supplementary teaching method for overcoming the limitations of high-fidelity simulators, which are currently actively used in nursing education.

In conclusion, an emotive role-play program focused on caring for high-risk pregnant women, delivered over a total of 11 hours over 3 days, was effective in improving nursing students’ communication skills and clinical performance; but no significant changes in emotional intelligence were found. Thus, emotive role-play is a feasible teaching mode that can be used to improve nursing students’ communication skills and clinical performance. Future studies that apply the program to clinical nurses who encounter high-risk pregnant women and examine its effectiveness may be helpful for identifying the utility of role-playing in clinical practice. Further research that is designed to reinforce emotional intelligence may also benefit emotive role-playing for nursing students.

## Figures and Tables

**Table 1.** Main contents of the emotive role-play program on high-risk pregnancy

Day	Session	Theme	Contents	Materials	Methods	Duration (minute)
1	1	Introduction	-Welcome and self-introduction	-Workbook	-Group building and sharing	30
			-Program introduction	-Participant pledge	-Writing the participant’s pledge	
	2	Preparation session 1: The importance of communication and communication methods	-The importance of communication between patients and nurses	-Workbook	-Lecture on communication by director of the Communication Center	150
			-Communication methods using cases of high-risk pregnancy and childbirth	-Painting tools	-Group presentation	
			-Group communication work		-Reflection journal	
2	3	Preparation session 2: Hearing the stories of women who have experienced high-risk pregnancy or childbirth	-Understanding and empathizing through stories about induced labor, preeclampsia, preterm premature rupture of membranes and preterm labor, and infertility	-Workbook	-Presentations of the experiences of four women	120
				-Reflection journal	-Reflection journal	
	4	Role-play training	-Empathizing, engaging emotions, and understanding and applying nursing performance through role-play scenarios involving induced labor, preeclampsia, preterm premature rupture of membranes and preterm labor, and infertility	-Workbook	-Review of eight different scenarios assigned to groups and discussion	120
				-Painting tools	-Select one case per group	
					-Revise the scenario script	
					-Practice role-play with an actress	
					-Making a role-play poster	
					-Reflection journal	
3	5	Performing and watching role-play scenarios	-Empathizing, engaging emotions, and understanding and applying nursing performance through role-play and watching performances	-Role-play props, stage installation, and auditorium preparation	-Role-play	180
					-Watching performances	
					-Making a pregnant woman’s body shape using balloons	
					-Mutual evaluation using “like” stickers	
					-Reflection journal	
	6	Evaluation and feedback	-Review previous session		-Reflecting on the role-play	60
			-Sharing and presentation of impressions		-Individual presentation	
			-Evaluation of role-play		-Reflection journal	
			-Conclusion			
Total						660

**Table 2.** Homogeneity of general characteristics and outcome variables between the experimental and comparison groups (N=83)

Variable	Categories	Mean±SD or n (%)		t or *χ*^2^	*p*
		Experimental(n=45)	Comparison (n=38)		
Age (year)		21.24±1.05	21.55±1.18	–1.26	.211
Sex	Male	1 (2.2)	4 (10.5)		.174^†^
	Female	44 (97.8)	34 (89.5)		
Religion	Yes	18 (40.0)	9 (23.7)	2.50	.114
	No	27 (60.0)	29 (76.3)		
Communication skills		4.01±0.41	4.08±0.49	–0.68	.499
Clinical performance		3.70±0.48	3.80±0.51	–0.85	.396
Emotional intelligence		5.32±0.74	5.24±0.83	0.51	.608

† Fisher exact test.

**Table 3.** Comparison of dependent variables between the experimental and comparison groups (N=83)

Variable	Group	Mean±SD			t	*p* (one-tailed)
		Pretest	Posttest	Mean differences (posttest–pretest)		
Communication skills	Experimental (n=45)	4.01±0.41	4.21±0.42	0.20±0.40	1.84	.035
	Comparison (n=38)	4.08±0.49	4.12±0.45	0.04±0.37		
Clinical performance	Experimental (n=45)	3.70±0.48	4.22±0.47	0.52±0.50	2.75	.004
	Comparison (n=38)	3.80±0.51	4.00±0.47	0.21±0.51		
Emotional intelligence	Experimental (n=45)	5.32±0.74	5.79±0.60	0.47±0.77	1.36	.088
	Comparison (n=38)	5.24±0.83	5.48±0.88	0.25±0.69		
